# Pharmacological effects and mechanisms of paeonol on antitumor and prevention of side effects of cancer therapy

**DOI:** 10.3389/fphar.2023.1194861

**Published:** 2023-06-20

**Authors:** Xindi Chang, Xiaoteng Feng, Min Du, Sijin Li, Jiarou Wang, Yiru Wang, Ping Liu

**Affiliations:** ^1^ Department of Cardiology, Longhua Hospital, Shanghai University of Traditional Chinese Medicine, Shanghai, China; ^2^ Longhua Hospital, Shanghai University of Traditional Chinese Medicine, Shanghai, China

**Keywords:** paeonol, cancer, side effects, mechanism, pharmacological effects

## Abstract

Cancer represents one of the leading causes of mortality worldwide. Conventional clinical treatments include radiation therapy, chemotherapy, immunotherapy, and targeted therapy. However, these treatments have inherent limitations, such as multidrug resistance and the induction of short- and long-term multiple organ damage, ultimately leading to a significant decrease in cancer survivors’ quality of life and life expectancy. Paeonol, a nature active compound derived from the root bark of the medicinal plant Paeonia suffruticosa, exhibits various pharmacological activities. Extensive research has demonstrated that paeonol exhibits substantial anticancer effects in various cancer, both *in vitro* and *in vivo*. Its underlying mechanisms involve the induction of apoptosis, the inhibition of cell proliferation, invasion and migration, angiogenesis, cell cycle arrest, autophagy, regulating tumor immunity and enhanced radiosensitivity, as well as the modulation of multiple signaling pathways, such as the PI3K/AKT and NF-κB signaling pathways. Additionally, paeonol can prevent adverse effects on the heart, liver, and kidneys induced by anticancer therapy. Despite numerous studies exploring paeonol’s therapeutic potential in cancer, no specific reviews have been conducted. Therefore, this review provides a systematic summary and analysis of paeonol’s anticancer effects, prevention of side effects, and the underlying mechanisms involved. This review aims to establish a theoretical basis for the adjunctive strategy of paeonol in cancer treatment, ultimately improving the survival rate and enhancing the quality of life for cancer patients.

## 1 Introduction

In recent times, the global incidence and mortality rates of cancer have exhibited a rapid upsurge, owing to several factors, including the progress of human society, economic development, and other multifarious factors. According to data, cancer is currently the second leading cause of death globally, following ischemic heart disease, and is projected to become the primary cause of death by 2060 (Bray et al., 2018; Mattiuzzi and Lippi, 2019). In particular, high-income countries experience high incidence rates of certain cancers, such as lung, breast, and prostate. At the same time, it also has a high incidence in middle and low-income countries due to the influence of many of the same risk factors, such as smoking and obesity (Torre et al., 2016). Currently, the primary treatment modalities for cancer include surgery, radiation therapy, and systemic therapy, such as chemotherapy, targeted therapy, hormone therapy, and immunotherapy (Miller et al., 2019). However, modern treatment strategies that improve the chances of surviving cancer come with a corresponding cost (Curigliano et al., 2016). Cancer patients may experience complications in multiple organs, such as the heart, liver, kidney, lung, and gastrointestinal tract, associated with cancer treatment, severely impacting their quality of life and reducing life expectancy (Kennedy and Salama, 2020; Yazbeck et al., 2022). Additionally, the rapid development of multidrug resistance to anticancer drugs by cancer cells presents a significant limitation to cancer treatment (Kolodny et al., 2018). Therefore, searching for effective antitumor drugs with fewer toxic side effects is a crucial area of focus in developing oncology drugs.

Over the past few years, there has been growing interest in natural products as potential alternatives for combating cancer progression (Zhang et al., 2022). Among them, paeonol, a phenolic compound derived from the root bark of the medicinal plant peony, has demonstrated promising pharmacological activities, including anti-inflammatory, analgesic, anti-cardiovascular disease, and neuroprotective effects (Zhang et al., 2019). In addition, an increasing number of studies have paid attention to the extensive anti-tumor activity of paeonol, which has a significant inhibitory effect on various types of cancer cells through multiple pathways and multiple targets (Chen et al., 2022b; Ding et al., 2022). Simultaneously, paeonol plays a certain protective role against multiorgan damage in addition to its anticancer activity (Ding et al., 2022). Thus, paeonol is considered a potentially safe and effective antitumor agent that could be used as an alternative or supplement to conventional cancer therapy (Wan et al., 2008; Wu et al., 2016a). This study comprehensively reviews the pharmacological mechanisms underlying paeonol’s antitumor effects and its potential for reducing multiorgan toxicity induced by cancer therapy. Consequently, this research contributes to a better understanding of the potential benefits of paeonol in tumor therapy and its synergistic reducing-toxicity effects to provide a reference for the further development and clinical application of paeonol in anticancer therapy.

## 2 Pharmacological properties of paeonol

### 2.1 Chemical properties of paeonol

**FIGURE 1:**
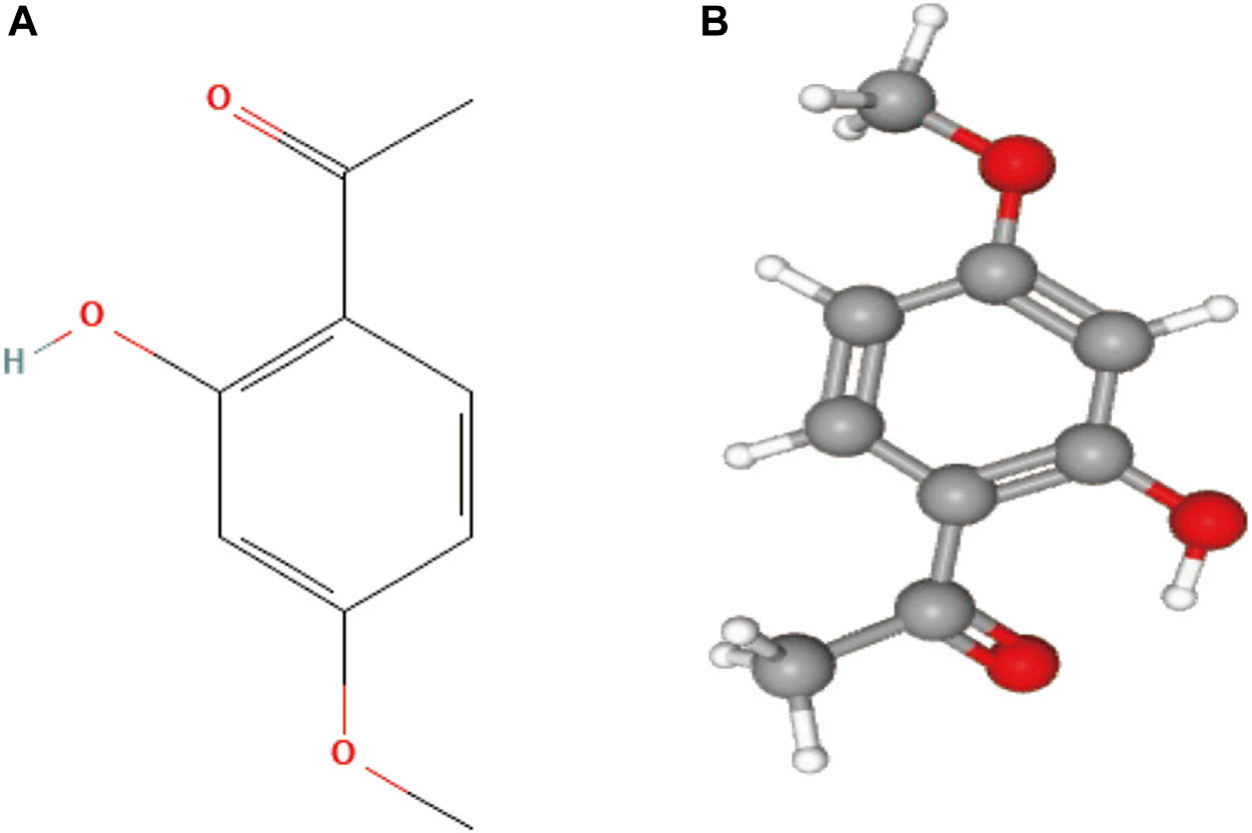
**(A)** 2D structure of paeonol, the molecular formula is C9H10O3, and PubChem CID is 11092; **(B)** 3D structure of paeonol.

Natural products possess unique, diverse, and complex structures, chemical properties, and a wide range of biological activities, making them increasingly attractive as potential drugs for treating various clinical diseases (Chen and Kirchmair, 2020). Paeonol is a natural active substance derived from the traditional Chinese medicine Cortex Moutan (CM). Modern analytical techniques have identified paeonol as the most abundant compound in CM and the key to its various pharmacological effects (Yang et al., 2020). Paeonol, chemically known as 2-hydroxy-4-methoxy acetophenone, and the chemical structures are presented in Figure 1. It has a low molecular weight of 166.17 g/mol and a melting point of 52°C (Latif et al., 2022). It has been found to exert antitumor effects through various molecular mechanisms (Fu et al., 2018; Cai et al., 2020; Zhang et al., 2020a). In addition, several derivatives of paeonol have been discovered and synthesized, which also exhibit high anticancer potential and provide structural templates for the design and development of new anticancer agents (Wang et al., 2012; Tsai et al., 2016).

### 2.2 Pharmacokinetics and new drug delivery systems of paeonol

The pharmacokinetics of paeonol in rats were found to be unsatisfactory after various routes of administration, exhibiting short peak concentration (Tmax) and elimination half-life (t1/2), as well as low oral bioavailability (only 16%) (Wu et al., 2003; Ma et al., 2008; Xie et al., 2008). The lower bioavailability of gavage administration compared to intravenous administration of paeonol may be attributed to incomplete absorption or a more pronounced first-pass effect (Ma et al., 2009). The rapid metabolism of paeonol in the heart, liver, kidney, and other tissues in the body seems to guarantee its safety because of the absence of long-term accumulation, but it also restricts its clinical utility (Hu et al., 2020). Moreover, paeonol exhibits drawbacks, such as low aqueous solubility, poor stability, and high volatility at room temperature. Therefore, extensive efforts have been made to develop various paeonol formulations and novel drug delivery systems, such as tablets, hydrogels, microparticles, microsponges, nanocapsules, polymer nanoparticles, nanospheres, microemulsions, and liposomes. These formulations not only enhance the solubility, chemical stability, and bioavailability of paeonol but also improve its pharmacological activity. Several studies have reported significant improvements in the efficacy and therapeutic potential of paeonol-based formulations (Ajazuddin and Saraf, 2010; Adki and Kulkarni, 2020).

A study reported the preparation of methoxy PEG-PCL (mPEG-PCL) nanoparticles as drug carriers to load paeonol (Pae-NPs), which showed more potent antitumor effects than free paeonol in both *in vitro* and *in vivo* experiments (Chen et al., 2017). Another study explored PEGylated liposome-mediated drug delivery systems for paeonol (Pae-PEG-NISVs) that effectively stabilize the delivery of the drug to cancer cells. This system has the potential to serve as an efficient carrier for paeonol, and a significant synergistic effect was observed with low concentrations of Pae-PEG-NISVs and 5-fluorouracil (5-Fu) (He et al., 2017). These results suggest that the novel drug delivery system of paeonol not only addresses the pharmacological limitations of paeonol but also enhances its antitumor efficacy, providing potential for the widespread application of paeonol.

## 3 Paeonol anticancer effects

### 3.1 Inhibition of cancer cell growth and proliferation

Dysregulation of cell proliferation is considered a significant contributing factor to various diseases. In particular, uncontrolled cell proliferation is a hallmark of cancer, and targeting this process is essential to controlling tumor progression (Loftus et al., 2022). There is considerable evidence to suggest that paeonol exhibits inhibitory effects on cancer cell growth and proliferation in a variety of cell lines, including cervix cancer HeLa cells (Du et al., 2022), human bladder cancer T24 and 5,637 cells (Zhang et al., 2021a), non-small-cell lung cancer A549 cells, hepatocellular carcinoma Hep3B, Huh-7, BEL-7404, SMMC-7721, MHCC97-H cells (Chunhu et al., 2008; Cai et al., 2020; Liu and Zhang, 2020), colorectal cancer HT-29 cells (Ye et al., 2009), pancreatic cancer Panc-1, Capan-1 cells (Cheng et al., 2020), osteosarcoma Saos-2, MG-63 cells (Zhou et al., 2020), ovarian cancer A2780, SKOV3, OVCAR-3 cells (Zhou et al., 2017; Gao et al., 2019), gastric cancer SGC-7901, BGC823, HGC-27 cells (Lyu et al., 2017; Fu et al., 2018), breast cancer MDA-MB-231 cells (Saahene et al., 2018), prostate cancer DU145, PC-3 cells (Xu et al., 2017), fibrosarcoma HT-1080 cells, esophageal cancer SEG-1, Eca-109 cells (Sun et al., 2008), glioma U87MG cells, U251 cells (Hao et al., 2023). These data indicate that paeonol has broad-spectrum antitumor properties, inhibiting tumor cell proliferation in a dose/time-dependent manner in various tumor cell lines. It was also found that paeonol exhibited more potent proliferation inhibition with 5-FU in a certain concentration range, while at higher concentrations, they did not show more significant synergistic effects (Chunhu et al., 2008). This interesting phenomenon shows that paeonol may exhibit synergistic or antagonistic effects with other chemotherapeutics at different concentrations, which may be related to the activation of the intrinsic defense mechanism of cells under the action of high concentrations of compounds to regulate and reduce the cytotoxicity of compounds. However, its mechanism needs to be further elucidated. In conclusion, paeonol has inhibitory effects on the growth of a variety of cancer cells *in vitro* and *in vivo*, and its effects are associated with the activation of cell apoptotic pathways and the regulation of the cell cycle.

### 3.2 Induction of apoptosis of cancer cells

Apoptosis is widely recognized as a form of programmed cell death critical in maintaining the normal growth and development of eukaryotes and homeostasis. Additionally, eliminating harmful or unnecessary cells in organisms is an essential mechanism for regulating cell death (Kaczanowski, 2016; Qian et al., 2022). Therefore, the disruption of apoptosis can lead to an imbalance of cell division and cell death, resulting in various diseases. In cancer, malignant cells can promote tumor growth and development by decreasing apoptosis or reducing resistance to apoptosis through multiple mechanisms (Wong, 2011). In several studies related to paeonol’s inhibition of tumors, significant apoptotic changes in tumor cells were clearly observed, which are typically characterized by cell shrinkage, chromatin accumulation and condensation, irregular nucleic shapes, and fragmentation of cells into membrane-wrapped apoptotic bodies, suggesting that the antitumor activity of paeonol is related to the mechanism of inducing apoptosis (Sun et al., 2008; Yin et al., 2013; Ou et al., 2014). There are two classical pathways of apoptosis, including the intrinsic (also known as the mitochondrial pathway) and extrinsic pathways (death receptor pathway), triggered by cellular stress, DNA damage, and immune surveillance mechanisms (Carneiro and El-Deiry, 2020). In the intrinsic apoptotic pathway, proapoptotic proteins (BAX and BAK) of the Bcl-2 family members promote cytochrome c release through mitochondrial membrane permeabilization and form apoptosomes with cysteine 9, which then activates the executioner cysteine 3 to promote apoptosis. In the extrinsic pathway, the death ligand binds to the TNF receptor superfamily to form a death-inducing signaling complex (DISC) composed of Fas-associated death domain (FADD) protein and proteinase 8, 10, thereby activating downstream effector caspases (caspase-3, 6, and 7), they are responsible for the execution phase of apoptosis (Su et al., 2015; Carneiro and El-Deiry, 2020). Previous studies have shown that paeonol significantly reduces the expression of Bcl-2, increases the expression of Bax, and reduces the ratio of Bcl-2/Bax, accompanied by the activation of caspase 3, 8 and 9 to promote apoptosis (Ou et al., 2014). When paeonol was combined with cisplatin, it showed a more substantial apoptosis-inducing effect (Wan et al., 2008). (Du et al., 2022; Li et al., 2014) reported that paeonol induces mitochondrial dysfunction, including the inducing of mitochondrial membrane potential (MMP), reactive oxygen species (ROS) production, and the release of cytochrome c. These results further suggest that paeonol mediates apoptosis through intrinsic and extrinsic pathways. In addition to the well-known apoptotic pathway, it is increasingly recognized that in different tumors concomitant disruption of endoplasmic reticulum (ER) homeostasis causes persistent ER stress (Chen et al., 2022b). Qin Niu demonstrated that paeonol promoted the expression of apoptotic proteins GRP78, CHOP, Caspase-12, and Caspase-3. It also promotes the release of Ca^2+^ from the endoplasmic reticulum into the cytoplasm, causing mitochondrial dysfunction and subsequently inducing apoptosis (Niu et al., 2020). However, it has been found that tumor cells can adapt to endoplasmic reticulum stress and thus evade apoptosis (Zhang et al., 2015b). In Lulu Fan’s study, it was found that increased expression of cyclooxygenase-2 (COX-2) may be critical for apoptosis resistance to endoplasmic reticulum stress, while paeonol downregulated COX-2 in hepatocellular carcinoma cells and reversed this effect (Fan et al., 2018).

Further research found that paeonol-mediated apoptosis involves multiple targets and pathways. Chen Yajing et al., based on a network pharmacology study, found that the expected targets in the biological process of paeonol and renal cell carcinoma (RCC) are mainly enriched in the positive regulation of cell death and apoptosis. The key signaling pathways mainly include the TNF signaling pathway, PI3K-AKT signaling pathway, and MAPK signaling pathway (Chen et al., 2022a). Activation of the PI3K-AKT signaling pathway and its downstream effector mTOR, leads to a cascade of cellular responses that promote cancer development. As a tumor suppressor, PTEN was found to play a crucial role in the negative regulation of the PI3K/AKT pathway (El-Tanani et al., 2023). The PTEN/PI3K/AKT/mTOR pathway is considered to be the key to the paeonol-induced apoptosis mechanism. Studies have found that paeonol can inhibit the phosphorylation expression of the PI3K/AKT pathway (Zhang et al., 2021; Du et al., 2022) and can inhibit the abnormal activation of this pathway by upregulating the level of PTEN (Zhang et al., 2006). MicroRNAs (miRNAs) are essential regulators of gene expression, and therefore miRNAs influence cancer progression by regulating the expression of oncogenes and oncogenes (Lai et al., 2019). A study found that miR-106a-5p is a critical upstream target for the regulation of PTEN and that paeonol can inhibit the expression of miR-106a-5p and negatively regulate PTEN expression, exerting proapoptotic effect (Zhang et al., 2021c). Nuclear factor κB (NF-κB) is also considered an essential target against cancer, and its activation promotes cancer cell proliferation and apoptosis inhibition (Chauhan et al., 2022). Similarly, paeonol has been shown to mediate apoptosis by inhibiting the NF-κB signaling pathway (Li et al., 2019). Jun Fu found that ERBB2 can activate the PI3K/AKT pathway, activating the downstream NF-κB, which was reversed by paeonol (Fu et al., 2018). The Wnt/β-catenin signal transduction pathway has been identified as a significant regulator of apoptosis. Paeonol can significantly reduce β-catenin and c-Myc protein expression, inhibiting the Wnt/β-catenin signaling pathway to play a proapoptotic role (Li et al., 2017). The chemokine CXCR3-B has also been found to induce proliferation inhibition and apoptosis. Studies have demonstrated that paeonol can modulate the expression of BACH1 and Nrf2 by mediating the CXCL4/CXCR3-B signaling pathway, resulting in the down-regulation of HO-1 and the promotion of cell apoptosis (Saahene et al., 2018). Nrf2 and HO-1 are recognized as essential antioxidants, and thus, paeonol may provide a novel idea for antitumor by modulating oxidative stress by inhibiting Nrf2/HO-1 expression (Mao et al., 2020). The above research shows that paeonol can play a role in promoting apoptosis through multiple pathways. Among these pathways, the intrinsic pathway appears to be particularly important for paeonol-induced apoptosis.

### 3.3 Inhibition of cancer cell invasion and metastasis

Secondary tumors, rather than primary tumors, are responsible for 90% of cancer deaths. This is because malignant cells have metastatic properties (Lambert et al., 2017). Primary malignant tumor cells can invade adjacent tissues, enter the vascular system, and spread throughout the body, causing severe secondary lesions (Novikov et al., 2021). This intricate process, known as the invasion-metastasis cascade, comprises five fundamental stages: invasion, intravasation, circulation, extravasation, and colonization (Fares et al., 2020; Babaei et al., 2021). The epithelial-mesenchymal transition (EMT) is widely recognized as the vital process of metastasis, which promotes the acquisition of malignant traits by cancer cells, while EMT is closely affected by extracellular matrix (EMC) hardness (Tian et al., 2022). Cell scratch-wound healing and transwell invasion assays provided the initial evidence that paeonol markedly suppressed cancer cell migration and invasion, which was associated with the inhibition of MMP2 and MMP9 of the matrix metalloproteinase (MMP) family because MMP can decompose EMC (Lyu et al., 2017). Moreover, the study revealed that paeonol effectively inhibited 5-lipoxygenase (5-LO), which stimulates cell invasion and migration by upregulating MMP2 and MMP9 expression (Sheng et al., 2021). MiRNAs participate in the regulation of various biological processes, including cell proliferation, differentiation, and apoptosis. Recently, miRNAs have also been discovered to directly target the core factors involved in epithelial-mesenchymal transition (EMT), contributing to cancer progression and metastasis (Chan and Wang, 2015). The study found that paeonol inhibited the EMT of cancer cells (E-cadherin expression was upregulated, while N-cadherin expression was downregulated), which was related to the regulation of miR-126–5p by paeonol and dependent on miR-126–5p to regulate its target genes ZEB2, a potential inducer of EMT, acts to suppress cell metastasis (Lv et al., 2022). Another study confirmed that paeonol upregulates miR-141 via the PKCδ and c-Src pathways to inhibit migration and invasion of cancer cells (Horng et al., 2014). Furthermore, paeonol’s inhibition of cell migration is inseparable from inhibiting the PI3K/AKT signaling pathway, and this effect can be reversed by upregulating miR-21 and downregulating miR-424–3p (Sun et al., 2020; Yang et al., 2022). Inflammatory cytokines and active substances in the tumor inflammatory microenvironment can activate various pathways that induce EMT and promote cancer cell migration (Tan et al., 2021). Several studies have shown that paeonol can reduce inflammation in the tumor microenvironment and inhibit cell migration by suppressing the STAT3/NF-κB and TGF-β1/Smad signaling pathways (Zhang et al., 2015a; Cheng et al., 2020; Zhang et al., 2020a). In summary, paeonol inhibits cell invasion and migration primarily by suppressing EMT, which involves the co-regulation of specific miRNAs and inflammatory signaling pathways.

### 3.4 Induce cancer cell cycle arrest

The progression of cancer is the result of cell division, and the cell cycle is a critical mechanism that regulates this process. By targeting cell cycle progression, it may be possible to inhibit cell proliferation and tumor growth (Matthews et al., 2022). The cell cycle is composed of four phases: Gap 1 (G1), DNA synthesis (S), Gap 2 (G2), and mitosis (M), and the progression of each phase is regulated by the activity of cyclins, cyclin-dependent kinases (CDK) (Goel et al., 2018; Suski et al., 2021). Studies have found that paeonol can arrest the cell cycle in the G0/G1 phase, affecting the DNA replication and synthesis of tumor cells and inhibiting cell proliferation (Li et al., 2013). Aberrant activation of the WNT/β-catenin signaling pathway has been reported to induce transcription of LEF/TCF-responsive genes (such as c-Myc, cyclin D1) involved in the cell cycle. It was further found that paeonol downregulates cyclin D1 and CDK4 and upregulates p21Cip1 to arrest the cell cycle in G0/G1 phase by inhibiting the activation of WNT/β-catenin signaling (Liu et al., 2020). In addition, paeonol has been found to stimulate the movement of gastric cancer and esophageal cancer cells from the G0/G1 phase to the S phase, increasing the proportion of cells in the S phase and fractions decreasing in the G0/G1 and G2/M phases, this effectively prevents the cells from progressing into the M phase (Sun et al., 2008a; Li et al., 2010). The G2/M phase is an essential phase of cell mitosis. In human melanoma cells, paeonol-induced cell arrest in the G2/M phase is dose-dependent (Tao et al., 2014). These results indicate that paeonol can arrest cancer cells in different cell cycles and thus inhibit cell proliferation. However, the relevant mechanism of paeonol’s cell specificity to the cell cycle remains unclear and needs further study.

### 3.5 Inhibition of angiogenesis

The process of developing from existing blood vessels to new capillaries is called angiogenesis, which is beneficial for wound healing and embryo development. However, when angiogenesis is abnormally accelerated, it provides essential oxygen and nutrients for tumor growth and metastasis, and this process is activated during tumor progression (Nishida et al., 2006; Rajabi and Mousa, 2017). This complex process is jointly regulated by its activator and inhibitor, and vascular endothelial growth factor (VEGF) is considered a crucial angiogenesis activator. Much experimental evidence indicates that VEGF is highly expressed in growing tumor tissue, promoting its growth, invasion, and metastasis compared to healthy tissue (Yang and Cao, 2022). Targeting VEGF may be a feasible approach to block tumor progression. Tang et al. (2014) discovered that paeonol inhibits VEGF expression in human glioblastoma cells in a time-dependent manner. Basic fibroblast growth factor (bFGF) also acts as an activator of angiogenesis and promotes angiogenesis by directly or indirectly acting to upregulate VEGF in endothelial cells (Przybylski, 2009). It was found that paeonol could inhibit the migration and angiogenesis of human umbilical vein endothelial cells (HUVECs) treated with bFGF. This effect was further found to be associated with the downregulation of Akt phosphorylation in bFGF-stimulated HUVECs by paeonol and a decrease in matrix metalloproteinase-2 and -9 expression in HT1080 human fibrosarcoma cells (Kim et al., 2009). These findings suggest that paeonol can target tumor angiogenesis by regulating PI3K/AKT signaling pathway and MMPs activity, thereby inhibiting cancer cell invasion and migration. The research team found that paeonol oxime (PO), a derivative of paeonol, also inhibited the angiogenesis of fibrosarcoma cells. Interestingly, PO was more lethal to tumor cells than normal cells, indicating that paeonol and its derivatives can target tumor cells to exert antitumor effects (Lee et al., 2010). However, the clinical application of angiogenesis inhibition is currently controversial, and few studies investigate the effects of paeonol on tumor angiogenesis. Therefore, further research is needed to clarify the complex mechanisms involved in tumor therapy and the angiogenesis of angiogenesis in tumor therapy.

### 3.6 Induction of autophagy of cancer cells

As we all know, autophagy is a conservative process of capturing and degrading damaged proteins and organelles. Autophagy delivers cytoplasmic components to lysosomes for degradation to maintain cell metabolism and cell homeostasis (Yun and Lee, 2018). It has been demonstrated that autophagy inhibits tumor growth by preventing tissue damage and inducing cell death through self-degradation pathways, particularly in the early stages of cancer (Li et al., 2020). In contrast, in advanced stages of cancer, accompanied by tremendous intrinsic tumor cell stress, autophagy is activated by cancer cells to maintain survival and regeneration (Onorati et al., 2018). Therefore, autophagy plays a dynamic role in tumors under the influence of multiple factors. In studies on the regulation of autophagy by paeonol, it exerted anticancer effects by upregulating autophagy-related genes Atg5, LC3-II, and Beclin1 proteins in colorectal cancer LoVo cells and inducing autophagy (Cheng et al., 2019). In another study, the antiproliferative and apoptosis-promoting effects of paeonol in ovarian cancer may be related to paeonol-induced cytoprotective autophagy, as evidenced by a significant increase in the expression of the autophagy marker LC3-II and a decrease in the expression of p62, accompanied by the accumulation of autophagosomes and autolysosomes, while inhibition of the Akt/mTOR pathway is paeonol-induced autophagy in ovarian cancer cells and induction of apoptosis key. The study further found that combining paeonol and autophagy inhibitors enhanced the antitumor activities in a xenograft nude mouse model (Gao et al., 2019). The regulation mechanism of autophagy activity suggests that combining autophagy inducers and autophagy inhibitors could be an effective strategy against cancer. Therefore, paeonol may be a promising candidate for inducing autophagy when combined with autophagy inhibitors for cancer treatment.

### 3.7 Modulation of tumor immunity

We know that conventional radiotherapy treatment regimens kill tumor cells while potentially destroying adjacent normal cells, affecting patient prognosis. Cancer immunotherapy harnesses the cytotoxic potential of the human immune system as an important strategy for treating malignant tumors (Zhang and Zhang, 2020). T cells are activated by binding T cell receptors (TCR) to major histocompatibility complex (MHC) molecules or short tumor antigen peptides presented by human leukocyte antigens, thus exerting a powerful tumor-killing capacity. Programmed cell death protein 1 (PD1) is an immune checkpoint receptor that inhibits T cell activation through its ligand 1 (PD-L1) and ligand 2 (PD-L2) interactions, leading to tumor cell escape from T cell-mediated induction (Sun et al., 2018). In the study of Xianjie Chen et al., the effect of paeonol on tumor immunomodulation was observed for the first time. It was found that tumor progression was accompanied by accelerated thymic atrophy and apoptosis of thymocytes (differentiated CD4^+^ and CD8^+^ T cells) and that paeonol increased the mRNA levels of granzyme B in thymocytes and inhibited melanoma growth. Paeonol targets miR-139e5p to downregulate PD1 expression in the thymus to abolish immune dysfunction in melanoma (Chen et al., 2023). This result suggests that paeonol has some immunomodulatory effects in combating tumor growth. However, the mechanisms involved in tumor immunity are relatively complex, and more research is still needed to explore whether paeonol also has immunomodulatory effects on other tumors and its underlying mechanism.

### 3.8 Reversing cancer chemotherapy resistance and enhancing radiosensitivity

Currently, there are various cancer treatment means, and chemotherapy as the first choice for cancer therapy has remarkable efficacy. However, cancer patients still have poor prognoses and high recurrence and metastasis rates. One of the main reasons is that tumor cells can produce phenotype resistance to drugs with different structures and functions (Gatti and Zunino, 2005). Cancer cells can act individually or in combination through various mechanisms such as DNA repair, enhanced drug inactivation and efflux, cell death inhibition, EMT, and tumor microenvironment (TM), leading to chemoresistance (Senthebane et al., 2017; Hasan et al., 2018). Almost all chemotherapy drugs used to treat cancer will produce drug resistance. *In vitro* and *in vivo* experiments have demonstrated that paeonol can reverse the resistance to chemotherapy drugs like paclitaxel, doxorubicin (DOX), apatinib, and cisplatin. Studies have found that paeonol reverses drug resistance by regulating drug efflux, specifically by targeting adenosine triphosphate binding cassette (ABC) transporter proteins and p-glycoprotein (P-gp), which may be associated with inhibition of SET-mediated PI3K/AKT signal pathway activity (Zhang et al., 2015b; Sun et al., 2016). Additionally, paeonol can also induce PTEN expression, further contributing to the reversal of drug resistance (Han et al., 2018). In addition, the downregulation of transgelin 2 expression by paeonol may be an additional target for reversing paclitaxel resistance (Cai et al., 2014). It is gradually recognized that the tumor microenvironment can promote chemoresistance by mediating endoplasmic reticulum stress (ERS). The research reveals that paeonol can rectify ER-induced drug resistance by targeting COX-2-mediated PI3K/AKT/CHOP pathway inactivation (Fan et al., 2013). In addition to being resistant to chemotherapy drugs, tumor cells often resist radiation, leading to tumor recurrence. Research has found that paeonol can promote cell apoptosis and enhance the sensitivity of cancer cells to radiation by inhibiting the expression of the PI3K/Akt pathway and its downstream proteins COX-2 and Survivin (Lei et al., 2013; Wu et al., 2014; Ren and Zhang, 2017). In response to the radiotherapy resistance induced by tumor hypoxia, paeonol plays a role by inhibiting hypoxia-inducible factor-1 (HIF-1) and its downstream target gene VEGF (Zhou et al., 2017). Thus, paeonol reverses chemoresistance and enhances radiosensitivity by promoting apoptosis, mediating ERS, inhibiting angiogenesis, and regulating drug efflux, which is closely related to the regulation of PI3K/AKT. Based on these findings, we concluded that paeonol could reverse chemoresistance and act as a safe radiosensitizer. In clinical application, the combination of paeonol with radiotherapy and chemotherapy may further enhance the anticancer efficacy.

## 4 Organ protective effect of paeonol on anticancer treatment-induced toxicity

### 4.1 Protective effects of paeonol on cardiotoxicity

Cancer treatments have undergone significant advancements in recent years, leading to improved patient survival rates. However, these treatments have been accompanied by adverse effects. In addition to conventional chemotherapy and radiotherapy, targeted therapy and immunotherapy may also result in short-term or long-term cardiac damage, such as cardiomyopathy, heart failure, arrhythmia, and hypertension, which have emerged as the second most common cause of morbidity and mortality among cancer survivors, after recurrent malignant tumors (Curigliano et al., 2016). DOX is an established chemotherapeutic drug for treating several types of cancers. However, its use is limited by its potential to induce cardiotoxicity in a dose-dependent and cumulative manner, which is linked to increased oxidative stress and lipid peroxidation, disruption of iron regulatory protein, perturbation of Ca^2+^ homeostasis, alterations in autophagy and apoptosis pathways, and the release of nitric oxide and inflammatory mediators (Shabalala et al., 2017; Rawat et al., 2021). A study was conducted to establish a model of DOX-induced cardiotoxicity using H9c2 cells *in vitro* and the adult zebrafish heart *in vivo*. The results showed that paeonol could inhibit apoptosis, hypertrophy, and fibrosis through activation of Notch1 signaling, thereby attenuating adriamycin-induced pathological cardiac remodeling to reverse cardiotoxicity (Thabassum Akhtar Iqbal et al., 2020). Mitochondria are responsible for providing most of the energy for cardiomyocytes, and mitochondrial fusion and fission regulate the dynamic formation and remodeling of the mitochondrial network (Varga et al., 2015). DOX was found to inhibit mitochondrial fusion and accelerate mitochondrial fission, promoting ROS production and mitochondrial dysfunction and inducing cardiac myocardial injury, which was reversed by paeonol. Therefore, a deeper investigation of the mechanism revealed that paeonol exerts cardioprotective effects by restoring Mfn2-mediated mitochondrial fusion and function, and PKCε may act as a direct target of paeonol to activate Stat3-Mfn2 signaling pathway and promote mitochondrial fusion (Ding et al., 2023). The role of oxidative and nitrosative stress and inflammatory responses has been demonstrated in the methotrexate (MTX)-induced cardiotoxicity model. Administration of paeonol treatment reduced NOX-2, MAD, and NO levels and increased GSH and SOD expression. Simultaneous inhibition of the TLR4/NF-KB pathway to attenuate the inflammatory response to improve oxidative stress has also been suggested as a possible mechanism for paeonol to attenuate MTX-induced cardiotoxicity (Al-Taher et al., 2020). MiRNAs play a crucial role in the pathogenesis of various heart diseases. Evidence suggests that paeonol improves cardiac function in a rat model of DOX-induced chronic heart failure by inhibiting miR-21-5P and upregulating the expression of miR-21-5P downstream target S-phase kinase-associated protein 2 (SKP2) to inhibit apoptosis (Chen et al., 2022a). In epirubicin (EPI)-induced cardiotoxicity, LC3-II/I, Beclin-1, and Atg 5 levels were significantly decreased, indicating that autophagic activity was significantly inhibited, whereas paeonol reversed EPI-induced inhibition of autophagy. Further study revealed that miR-1 was downregulated in EPI-induced myocardial injury, while paeonol could inhibit the activation of PI3K/AKT/mTOR and its downstream NF-kB signaling pathway by increasing miR-1 expression, thus promoting autophagy and ameliorating myocardial injury (Wu et al., 2018). Our findings indicate that paeonol modulates oxidative stress, mitochondrial function, autophagy, apoptosis, and other mechanisms to regulate cardiotoxicity induced by anticancer treatments while synergistically enhancing anticancer effectiveness.

### 4.2 Protective effects of paeonol on hepatotoxicity

The liver is the primary organ responsible for drug metabolism. Therefore, chemotherapy treatments can potentially cause reversible or permanent damage to the liver, such as steatosis, steatohepatitis, and sinusoidal obstruction (White et al., 2016). Additionally, patients with pre-existing liver disease or liver cancer may experience aggravated hepatotoxicity (Thatishetty et al., 2013). *In vivo* and *in vitro* studies, EPI can cause hepatocyte death, while co-treatment of EPI and paeonol can decrease hepatocytes death and reverse the increased levels of hepatic functional enzymes (ALT, AST, ALP, and GGT) while attenuating EPI-induced oxidative stress injury (SOD, CAT, and GSH) and inflammatory response (IL-2, IL-6, TNF-α, IFN-α), which is related to the inhibition of PI3K/AKT/NF-kB pathway (Wu et al., 2016b). The research team utilized metabonomics research to discover that paeonol effectively inhibits autophagy induced by the AMPK/mTOR signaling pathway. Its metabolic mechanism involves intricate interactions with lipid metabolism (specifically palmitic acid and oleic acid), amino acid metabolism (serine, valine, leucine, and glycine), and energy metabolism (succinate and malate) (Jing et al., 2019). In MTX-induced hepatotoxicity model in mice, paeonol has been shown to promote the expression of drug efflux transporters, specifically P-gp and multidrug resistance-associated protein 2 (Mrp-2). This mechanism may be vital in reversing the hepatotoxicity caused by MTX hepatic accumulation (Morsy et al., 2022a). These findings offer significant contributions to the comprehension of the hepatoprotective properties of paeonol via the regulation of metabolic pathways. These outcomes emphasize the clinical potential of paeonol in preventing and treating hepatotoxicity.

### 4.3 Protective effects of paeonol on nephrotoxicity

The kidney serves as the primary pathway for eliminating both anticancer drugs and their metabolites, leading to damage of glomeruli and renal tubules during the excretion of chemotherapy drugs. In addition to conventional cytotoxic drugs, molecularly targeted drugs have also been implicated in causing nephrotoxicity, manifesting as a range of kidney diseases and electrolyte imbalances (Małyszko et al., 2016). Cisplatin is a widely used chemotherapeutic drug that is known to cause nephrotoxicity. A mouse model of cisplatin-induced acute renal failure showed signs of renal tubular injury with elevated serum creatinine and urea levels. In comparison, paeonol reversed these results and attenuated cisplatin-induced high levels of pro-inflammatory cytokines (TNF-α and IL-1β) and nitrite. These results suggest that the protective effect of paeonol against cisplatin-induced nephrotoxicity may be mediated by inhibiting inflammation and nitrosative stress (Lee et al., 2013). (Wu et al., 2017) further discovered that paeonol protects against DOX-induced nephrotoxicity by activating the Nrf2/HO-1 pathway to mitigate oxidative stress and nitrosative stress and by suppressing the inflammatory response through the NF-κB pathway. Moreover, paeonol attenuating the nephrotoxicity associated with renal accumulation of MTX by promoting the expression of renal efflux transport proteins is an important mechanism (Morsy et al., 2022b). A recent study has revealed that overexpression of the long non-coding RNA (lncRNA) maternally expressed gene 3 (lnc-MEG3) can lead to the inhibition of miRNA-126 and the subsequent activation of the AKT/TSC/mTOR pathway, which in turn suppresses autophagy and aggravates cisplatin-induced nephrotoxicity. Instead, paeonol can target and inhibit lnc-MEG3 and its related signaling pathways to regulate autophagy, exerting nephroprotective effects (Jing et al., 2021). These findings suggest that lncRNA or miRNA may be novel targets for preventing cancer treatment-induced nephrotoxicity, and the underlying mechanisms need to be thoroughly investigated.

### 4.4 Protective effects of paeonol on testicular injury

The administration of cytotoxic chemotherapy and radiotherapy as therapeutic interventions in malignancies, such as lymphoma, testicular cancer, osteosarcoma, and lung cancer, can elicit testicular dysfunction in male patients as a prevalent and significant long-term adverse effect. The resultant dysfunction manifests as either temporary or permanent oligospermia and azoospermia, owing to the detrimental effects of these therapies on the germinal epithelium (Howell and Shalet, 2005). The study revealed that paeonol significantly ameliorated MTX-induced testicular damage, as evidenced by the augmentation of testicular weight, elevation of testosterone levels, and improvement in pathological changes within the seminiferous tubules. The underlying mechanisms of paeonol’s protective effects are attributed to its ability to upregulate testicular P-gp levels and downregulate TNF-α and caspase 3 expression, concurrent with a decline in MDA and NOx levels and a rise in GSH levels and SOD activity. It can be surmised that paeonol protects against MTX-induced testicular injury by mitigating oxidative stress, inflammation, and apoptosis. Furthermore, the upregulation of P-gp expression mediated by paeonol is a promising and effective protective mechanism against MTX-induced testicular injury (Morsy et al., 2020).

## 5 Discussion and future perspectives

**FIGURE 2:**
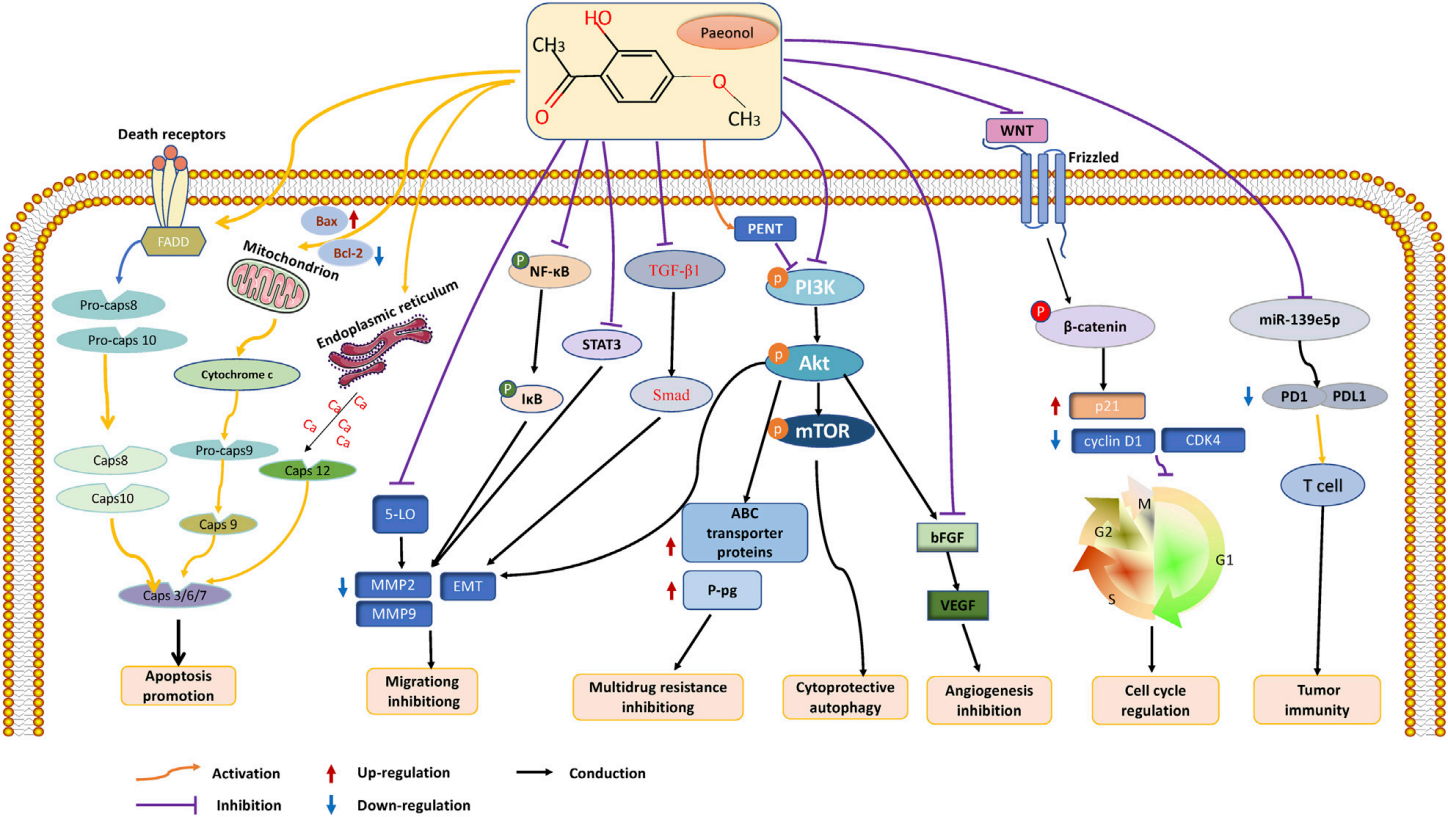
The anticancer effects and mechanisms of paeonol. Paeonol can exert anticancer effects through various mechanisms, including 1. Inhibiting the proliferation of various tumor cells; 2. Inhibit cell invasion and migration by inhibiting EMT of tumor cells; 3. Promote intracellular apoptosis through intrinsic and extrinsic pathways; 3. Induce cell cycle arrest, including G1, S, G2, and M phases; 4. Inhibit angiogenesis by targeting VEGF; 5. Promote the formation of autophagosomes and autophagy of cancer cells; 6. Regulating tumor immunity; 7. Reverse multidrug resistance and increase radiosensitivity.

With the continuous progress of medical diagnosis and treatment, the mortality rate of cancer is showing a downward trend year by year (Siegel et al., 2023). However, the acute or chronic toxic side effects caused by various anticancer therapies seriously affect patients’ quality of life (Schirrmacher, 2019). Therefore, exploring natural products with abundant anticancer potential has become the focus of cancer drug development (Huang et al., 2021). Paeonol, a bioactive substance found in CM, has demonstrated excellent effectiveness against malignant tumors in both *in vitro* and *in vivo* experiments. Its anticancer functions include inhibiting tumor growth, protecting against multiorgan damage caused by cancer treatment, and demonstrating broad-spectrum antitumor effects that inhibit a wide range of tumor cells (including lung cancer, stomach cancer, breast cancer, prostate cancer, and many other cancers). Paeonol’s numerous pharmacological activities enable it to act through various mechanisms, including the induction of cancer cell apoptosis, autophagy, cell cycle arrest, and inhibition of cancer cell migration and angiogenesis. These mechanisms implicate the participation of various molecular targets and signaling pathways, among which the PI3K/AKT/mTOR and NF-κB pathways are significant. Furthermore, miRNAs can potentially be a promising target for studying the anticancer effects of paeonol. (Figure 2).

**FIGURE 3:**
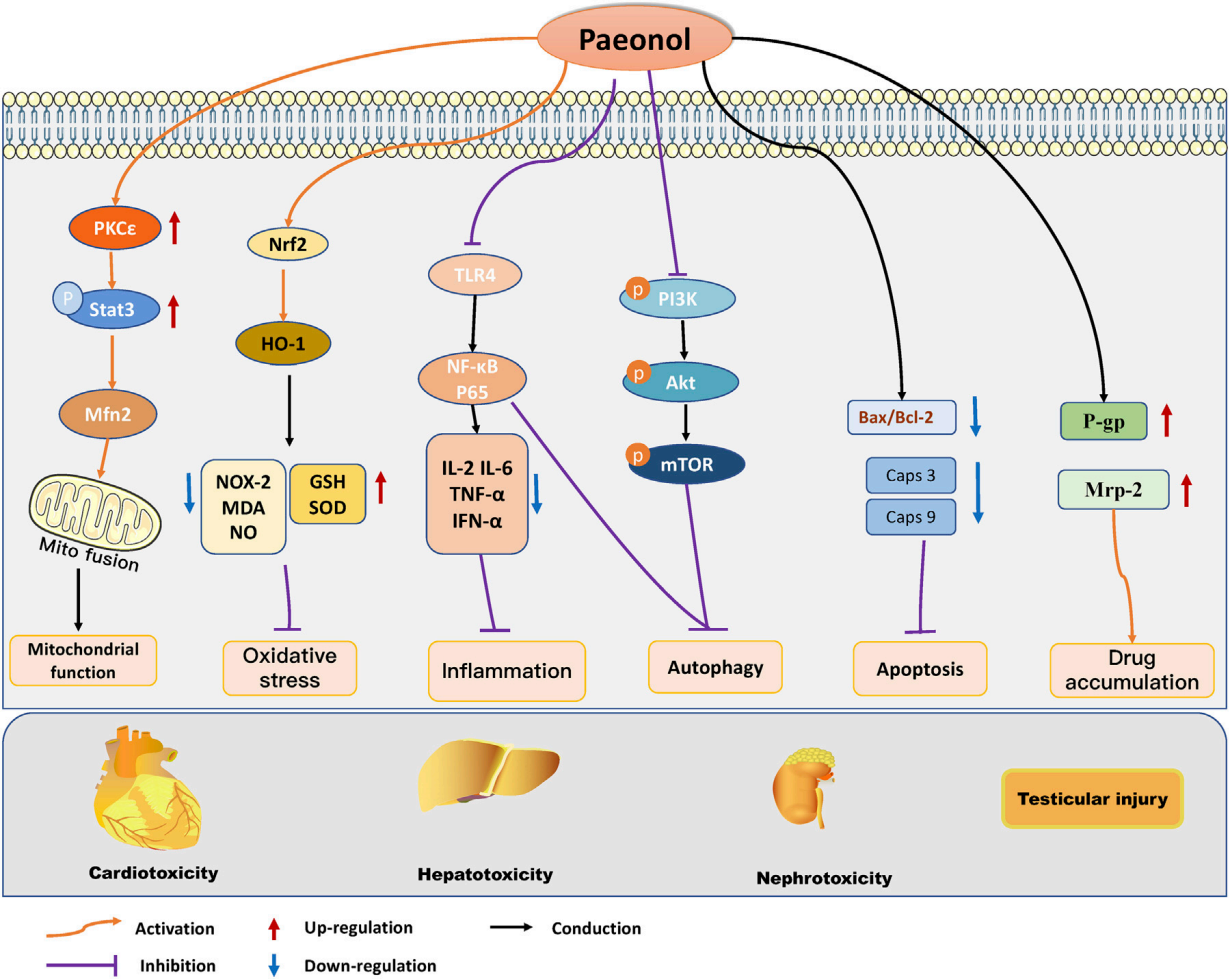
Molecular protective mechanism of paeonol against cancer therapy-induced side effects, mainly including cardiotoxicity, hepatotoxicity, nephrotoxicity, and testicular damage. Paeonol modulates oxidative stress, mitochondrial function, autophagy, apoptosis, inflammation, and apoptosis to regulate multiple organ injuries induced by anticancer treatments.

The multiorgan toxicities associated with cancer therapeutics, including those affecting the heart, liver, kidneys, and testes, represent a significant impediment to the clinical application of cancer therapeutics and can ultimately impact the prognosis of cancer patients. Paeonol was found to have a protective effect on multiorgan damage induced by anticancer treatment. Research has revealed that paeonol can ameliorate heart, liver, kidney, and testicular injury caused by chemotherapy drugs such as DOX, EPI, and MTX. This is accomplished by regulating oxidative stress, mitochondrial function, inflammatory reaction, autophagy, and cell apoptosis. (Figure 3). Combined with the existing studies, we found that paeonol has significant anticancer activity and certain organ protective effects, which may be related to the fact that paeonol exerts different regulatory mechanisms in different cell types. For example, in tumor cells, paeonol can induce apoptosis and inhibit tumor growth, while under the intervention of chemotherapeutic drugs, cardiomyocytes, hepatocytes, and renal cells showed noticeable apoptotic changes, at which time paeonol can exert organ-protective effects by inhibiting apoptosis. In addition, paeonol plays an essential role in reversing chemotherapy drug resistance and organ damage induced by anticancer treatment by modulating drug efflux. However, it was found that paeonol could reverse paclitaxel resistance by down-regulating P-gp expression. Still, it can protect against organ damage by upregulating P-gp expression in chemotherapeutic drug-induced liver toxicity and testicular injury. This suggests that regulating P-gp levels by paeonol in malignant and non-malignant cells may be multifactorial, and the mechanism needs further investigation. Gastrointestinal toxicity is another common side effect associated with anticancer treatment, and in severe cases, it can be life-threatening (Huang et al., 2021). However, to date, no research has been conducted on the potential benefits of paeonol in combating gastrointestinal toxicity, indicating a promising avenue for future investigation.

To overcome the limitations associated with the poor physical properties of paeonol, including limited solubility, poor stability, rapid metabolism, and low bioavailability, various drug delivery systems have been developed, such as nanocarriers, liposomes, and microemulsions. These drug delivery systems have not only effectively improved the pharmacokinetic deficiencies of paeonol but also enhanced its target specificity, resulting in increased bioavailability and anticancer efficacy of paeonol, thereby addressing the practical limitations of its application.

In conclusion, paeonol exerts antitumor activity by inhibiting cell proliferation, inducing apoptosis, inhibiting metastasis and angiogenesis, regulating tumor immunity, reversing drug resistance, and protecting against cancer treatment-induced multiorgan damage by regulating oxidative stress, apoptosis, mitochondrial function, and autophagy. It is a promising natural active medicine. Combined with the dual regulatory effects of paeonol in tumors, we have reason to believe that paeonol may have a wide range of prospects as an adjuvant strategy or even as a primary therapy in clinical treatment. However, the current research on the effects of paeonol on tumor prevention and therapy mainly comprises basic experimental studies and lacks clinical evidence to support these findings. Therefore, large-scale, high-quality, randomized controlled trials are necessary to evaluate the safety and efficacy of paeonol in clinical tumor patients and provide the clinical data needed to support the widespread use of paeonol.

## Figures and Tables

**Table:** 

**CM**	Cortex Moutan
**5-Fu**	5-Fluorouracil
**Bax**	Bcl2-associated X
**Bcl-2**	B-cell lymphoma-2
**PI3K**	Phosphatidyl-inositol 3-kinase
**AKT**	Serine-threonine kinase
**NF-κB**	Nuclear factor kappa-B
**PTEN**	Phosphatase and tensin homolog
**COX-2**	Cyclooxygenase-2
**PGE2**	Prostaglandin E2
**IAPs**	Inhibitor of apoptosis proteins
**XIAP**	X-linked inhibitor of apoptosis protein
**c-IAP1**	Cellular Inhibitor of Apoptosis Protein 1
**CXCR3**	Chemokine (C-X-C motif) receptor 3
**CXCL4**	Platelet factor 4
**BACH1**	BTB and CNC homology 1
**Nrf2**	Nuclear factor erythroid 2-related factor 2
**HO-1**	Heme oxygenase 1
**ER**	Endoplasmic reticulum
**GRP78**	Glucose-regulated protein 78
**CHOP**	C/EBP-homologous protein
**EMT**	Epithelial-mesenchymal transition
**EMC**	Extracellular matrix
**MMP**	Metalloproteinase
**5-LO**	5-lipoxygenase
**miRNAs**	MicroRNAs
**EMT**	Epithelial-mesenchymal transition
**ZEB2**	Zinc finger E-box-binding homeobox 2
**PKCδ**	Protein kinase C-delta
**c-Src**	Proto-oncogene tyrosine-protein kinase Src
**STAT3**	Signal transducer and activator of transcription 3
**TGF-β**	Transforming growth factor Beta
**CDK**	Cyclin-dependent kinases
**VEGF**	Vascular endothelial growth factor
**bFGF**	Basic fibroblast growth factor
**PO**	Paeonol oxime
**TM**	Tumor microenvironment
**ABC**	Adenosine triphosphate binding cassette
**P-gp**	P-glycoprotein
**CHOP**	C/EBP homologous protein
**HIF-1**	Hypoxia inducible factor-1
**DOX**	Doxorubicin
**PKCε**	Protein kinase C epsilon type
**STAT3**	Signal transducer and activator of transcription 3
**ROS**	Reactive oxygen species
**EPI**	Epirubicin
**ALT**	Alanine aminotransferase
**AST**	Aspartate aminotransferase
**ALP**	Alkaline phosphatase
**GGT**	Gamma-glutamyl transferase
**SOD**	Auperoxide dismutase
**CAT**	Catalase
**GSH**	Glutathione
**IL-2**	Interleukin-2
**IL-6**	Interleukin 6
**TNF-α**	Tumor necrosis factor-alpha
**IFN-α**	Interferon-α
**AMPK**	AMP-activated protein kinase
**MTX**	Methotrexate
**P-gp**	P-glycoprotein
**IL-1β**	Interleukin-1 beta
**lncRNA**	Long non-coding RNA
**MDA**	Malondialdehyde
**NOx**	Nitric oxide
**GSH**	Glutathione
**mTOR**	Mammalian target of rapamycin
